# Using a theory-based, customized video game as an educational tool to improve physicians’ trauma triage decisions: study protocol for a randomized cluster trial

**DOI:** 10.1186/s13063-024-07961-w

**Published:** 2024-02-16

**Authors:** Deepika Mohan, Derek C. Angus, Chung-Chou H. Chang, Jonathan Elmer, Baruch Fischhoff, Kim J. Rak, Jacqueline L. Barnes, Andrew B. Peitzman, Douglas B. White

**Affiliations:** 1 Department of Surgery, University of Pittsburgh School of Medicine, F1265 PUH, 200 Lothrop St, Pittsburgh, PA 15213 USA; 2 Department of Critical Care Medicine, University of Pittsburgh School of Medicine, Pittsburgh, PA USA; 3 Department of Emergency Medicine, University of Pittsburgh School of Medicine, Pittsburgh, PA USA; 4 Department of Neurology, University of Pittsburgh School of Medicine, Pittsburgh, PA USA; 5 Department of Engineering and Environmental Policy, Carnegie Mellon University, Pittsburgh, PA USA

**Keywords:** Trauma triage, Clinical practice guidelines, Physicians, Heuristics, Deliberate practice, Diagnostic skill, Randomized controlled trial

## Abstract

**Background:**

Transfer of severely injured patients to trauma centers, either directly from the field or after evaluation at non-trauma centers, reduces preventable morbidity and mortality. Failure to transfer these patients appropriately (i.e., under-triage) remains common, and occurs in part because physicians at non-trauma centers make diagnostic errors when evaluating the severity of patients’ injuries. We developed *Night Shift*, a theory-based adventure video game, to recalibrate physician heuristics (intuitive judgments) in trauma triage and established its efficacy in the laboratory. We plan a type 1 hybrid effectiveness-implementation trial to determine whether the game changes physician triage decisions in real-life and hypothesize that it will reduce the proportion of patients under-triaged.

**Methods:**

We will recruit 800 physicians who work in the emergency departments (EDs) of non-trauma centers in the US and will randomize them to the game (intervention) or to usual education and training (control). We will ask those in the intervention group to play *Night Shift* for 2 h within 2 weeks of enrollment and again for 20 min at quarterly intervals. Those in the control group will receive only usual education (i.e., nothing supplemental). We will then assess physicians’ triage practices for older, severely injured adults in the 1-year following enrollment, using Medicare claims, and will compare under-triage (primary outcome), 30-day mortality and re-admissions, functional independence, and over-triage between the two groups. We will evaluate contextual factors influencing reach, adoption, implementation, and maintenance with interviews of a subset of trial participants (*n* = 20) and of other key decision makers (e.g., patients, first responders, administrators [*n* = 100]).

**Discussion:**

The results of the trial will inform future efforts to improve the implementation of clinical practice guidelines in trauma triage and will provide deeper understanding of effective strategies to reduce diagnostic errors during time-sensitive decision making.

**Trial registration:**

ClinicalTrials.gov; NCT06063434. Registered 26 September 2023.

**Supplementary Information:**

The online version contains supplementary material available at 10.1186/s13063-024-07961-w.

## Introduction

### Background and rationale

Injury is the leading cause of loss of independence among those over the age of 65, resulting in ≥ 3 million emergency department (ED) visits, ≥ 800,000 hospitalizations, and ≥ $50 billion in costs each year [1]. The appropriate triage of trauma patients, defined as the rapid identification of those with severe injuries and transfer to trauma centers either directly from the field or after evaluation at a non-trauma center, decreases mortality by 10–25%, reduces loss of independence, and diminishes pain at 1 year [2–6]. Consequently, stakeholders have implemented clinical practice guidelines for trauma triage using best-practice methods such as text-based education, outreach by opinion leaders, and legislative mandates [3]. Despite these efforts, under-triage at non-trauma centers remains common (~ 50–80%), particularly among older adults [7–9].

Physicians are the largest source of non-compliance with clinical practice guidelines [10]. Experimental evidence from the basic behavioral sciences literature and our prior research suggests that people typically rely on intuitive judgments (heuristics) to make complex decisions under pressure, as in the case of trauma triage [11–13]. When calibrated well, heuristics allow rapid, accurate decisions [14]. When calibrated poorly, they produce errors in diagnoses [15]. We previously developed a customized, theory-based video game (*Night Shift*) to recalibrate physician heuristics in trauma triage, using the platform of an adventure game to train physicians to use clinical practice guidelines by making them relevant and memorable. In pilot trials, physicians who played the game made 10–18% more guideline-concordant decisions on a validated virtual simulation compared with those who completed a gold-standard, text-based educational program, an effect that persisted through the 6-month follow-up [16, 17].

### Objectives and trial design

The objective of this type 1 effectiveness-implementation trial is to evaluate the effect of *Night Shift* on real-world triage decision making and on patient outcomes. We include a trial schematic in eFigure 1 and the SPIRIT checklist in the supplemental materials. We will randomize a national sample of physicians who work at non-trauma centers in the US (*N* = 800) to play a video game (*Night Shift*) or to usual education (control) and will use Medicare claims data to evaluate the groups’ triage practices for severely injured patients who present initially at non-trauma centers in the 1 year after enrollment in the trial. We will subsequently conduct a series of semi-structured interviews with trial participants and with key decision makers (e.g., ED directors, paramedics [*N* = 100]) to identify contextual factors that would influence implementation of the intervention in the future. We hypothesize that physicians randomized to play the video game will under-triage a smaller proportion of severely injured patients compared to those randomized to the control (usual education) group. We secondarily hypothesize that intervention physicians will have fewer adverse patient outcomes (e.g., 30-day mortality and 30-day readmissions) compared to physicians in the control group.

## Methods

### Study setting, eligibility criteria, recruitment, and consent procedures

We have partnered with 3 US (United States) physician staffing groups. Cumulatively, these groups employ approximately 4500 physicians, cover ≥ 30 states, staff ≥ 600 EDs, and provide care to ≥ 16 million patients each year. We plan to recruit board-certified physicians who work exclusively in the EDs of non-trauma centers in the US, who triage adult trauma patients as part of their practice, and who have a National Provider Identifier (NPI). We plan to exclude non-physician healthcare professionals (e.g., nurse practitioners, physician assistants) because of variation in billing practices (e.g., some bill under their own identifiers while others do not) that will confound outcome assessment. We will also exclude physicians who work at both trauma and non-trauma centers (because this limits the number of eligible patients they encounter), and those who work outside the continental US (because of differences in referral patterns). We will ask physician leaders of the organizations, with which we have partnered, to distribute an email to their staff that describes the trial, and includes a link to the consent form. Physicians who provide consent will then receive a survey that collects demographic data that will allow us to assess eligibility.

### Additional consent provisions for collection and use of participant data and biological specimens

We are not planning any ancillary studies and therefore have not outlined any additional consent provisions.

### Interventions

#### Explanation for the choice of comparator

Stakeholders in trauma, including the American College of Surgeons and the American Board of Emergency Medicine (ABEM), have already executed best-practice educational efforts to increase the implementation of trauma triage guidelines, including widespread dissemination of the guidelines through Advanced Trauma Life Support (ATLS), a 2-day textbook-based course completed quadrennially by > 80% of physicians who work in non-trauma centers, and a 4-h trauma resuscitation module required quinquennially as part of the ABEM’s recertification program [18, 19]. We therefore consider usual education to be the best comparator for our intervention.

#### Intervention description

*Night Shift* is an adventure video game designed to recalibrate physician heuristics for identifying severely injured trauma patients (i.e., their pattern recognition) that we developed originally in 2016. Players take on the persona of Andy Jordan, a young emergency medicine physician, who moves home to search for his missing grandfather, and takes a job at a small community hospital. The player must not only solve the mystery but also manage a series of trauma and non-trauma cases, experiencing the consequences of his/her decision making. Based on feedback from participants in laboratory-based trials, we partnered with Schell Games (Pittsburgh, PA) to modify the user interface, simplifying the movement controls and refining the clinical content. We also expanded the game to allow for its longitudinal use. Notably, we embedded a puzzle mini-game (*Graveyard Shift*) with levels that unlock at pre-specified intervals (e.g., levels 1–3 become available in March 2024; levels 4–6 become available in June 2024; levels 7–10 become available in September 2024), with the objective of encouraging participants to return to the game for booster sessions. The revised application has the name *Night Shift 2024* and will be available for download on the iOS application store. We summarize the theoretical framework, game content, and game mechanics of *Night Shift 2024* in Table 1 and share a schematic of the process that we followed to ensure theory-based development in eFigure 2.

**Table 1:** Description of *Night Shift 2024*

*Duration:* 3 + h of gameplay possible
*Objective:* To increase implementation of clinical practice guidelines in trauma triage
*Behavioral problem*: Diagnostic errors result in non-compliance with guidelines
*Theory of behavior:* The dual process model of cognition describes judgment (i.e., diagnosis) as the product of heuristics (system 1 processes) and rule-based algorithms (system 2 processes. System 1 processes allow people to solve difficult questions under conditions of time-pressure and uncertainty by providing solutions based on pattern recognition. System 2 processes require effort but provide more accurate answers from rule-based algorithms
*Methods of behavior change:* We selected 5 behavior change techniques from the taxonomy published by Michie et al.: demonstration of the behavior; increasing the salience of consequences of behavior; shaping knowledge; providing feedback on behavior, and offering opportunities to practice the behavior. We selected 4 methods of stimulating engagement from a review of the literature: realism, interest, identification, and transportation
*Game concept:* The player takes on the role of Andy Jordan, a young emergency medicine physician, who moves home after his grandfather’s disappearance and accepts a job at a local community hospital covering night shifts. Andy encounters a series of patients at the local hospital and must make diagnostic and therapeutic decisions for each case. The player receives feedback on his/her decision making from a variety of in-game characters. Concurrently, the player must resolve the mystery of Andy’s grandfather’s disappearance, which offers the player the opportunity to learn more about the character and the world he inhabits. After completing the main story arc, the player uncovers an embedded mini game (*Graveyard Shift*), which uses a series of short puzzles to reinforce the triage principles. Each puzzle includes a 5 step-game loop: triage of 10 cases over 90 s, structured case comparison, feedback, structured debriefing, review of the literature
*Game content:* • *Night Shift* includes 5 teaching trauma cases of patients with injuries frequently under-triaged at non-trauma centers (e.g., multi-system injuries). These cases play out longitudinally so that the player experiences the natural consequences of behavior and receives feedback (opprobrium or approval) on their decision making. We also included 2 non-teaching trauma cases, where patients decompensate and players must rescue them, to stimulate realism and interest. Finally, the game has 5 diagnostically challenging cases intended to challenge players • *Graveyard Shift* has 10 levels, each covering a different decision principle: o Severe injuries belong at trauma centers; markers for severe injuries include shock, intubation, mangled extremities, penetrating injuries to the torso or proximal extremities, paralysis, and multi-system injuries o Moderate injuries in the setting of diminished physiologic reserve belong at trauma centers; markers for diminished physiologic reserve include age > 70 and evidence of frailty o Hospitals that lack resources should transfer all patients with more than minor injuries o Minor injuries never require transfer to trauma centers
*Game mechanics:* The user interface includes tap-to-act, connect-the dots, points that unlock in-game rewards (e.g., the opportunity to learn more about the character), music, and time-pressure

We will ask participants to spend a minimum of 2 h playing *Night Shift 2024* within 14 days of receiving their device and then return to the game for 20 min at 90, 180, and 270 days after enrollment. Participants have the option of not completing the assigned study task, but we do not have pre-specified criteria for discontinuing or modifying the allocated intervention.

#### Strategies to improve adherence to the intervention and retention and to complete follow-up

We will pre-load new iPads with *Night Shift 2024* and will mail the devices to those allocated to the intervention group. Participants will keep their iPad as a fixed honorarium (approximate value: $350) and will also receive a conditional monetary honorarium for each booster dose that they complete ($25/session). They can also apply for 3 h of continuing medical education credit after completing all three booster sessions. We will issue three email reminders and a phone call during the first 2 weeks after enrollment and then quarterly reminders during the trial period. Participants in the usual education group will complete outcome assessment tools and will receive a conditional, wage-based honorarium ($100/hour spent) upon completion of study tasks. They too will receive email reminders and a phone call to encourage retention.

#### Relevant concomitant care permitted or prohibited during the trial and provisions for post-trial care

Participants may receive routine continuing medical education during the trial, although we have no mechanism in place to track that information. We have not made any provisions for post-trial care as we consider the likelihood of any harm extremely unlikely.

#### Outcomes

We will use the RE-AIM (*R*each, *E*ffectiveness, *A*doption, *I*mplementation, *M*aintenance) framework to evaluate this type 1 hybrid effectiveness-implementation trial and summarize the outcomes in.Table 2 [20, 21].

**Table 2:** Application of RE-AIM framework to trial outcomes. Our primary outcome measure is italicized

Domain and description	Name of measure and definition	Source of data	Level
**Reach** Absolute number, proportion, and representativeness of individuals who are willing to enroll in the trial	▪ Rate of response (# responded/# invited) ▪ Demographics of responders v. eligible	▪ Staffing organization information ▪ Participant surveys	▪ Staffing organization ▪ Physician
**Effectiveness** Impact of the intervention on behavioral and patient-centered outcomes	▪ *Under-triage* (1- proportion of severely injured patients transferred to a trauma center) ▪ Over-triage (proportion of patients transferred with minor injuries) ▪ 30-day mortality and readmissions (composite) ▪ Functional dependence (proportion of patients with 90-day pre-admission location at home with discharge to rehab or SNF)	▪ Medicare FFS and Advantage Professional, Outpatient, and Inpatient claims ▪ AHA and TIEP databases	▪ Physician ▪ Patient
**Adoption** Absolute number, proportion, and representativeness of settings	▪ Number, proportion, and characteristics of hospitals staffed by trial physicians v. acute care non-trauma centers	▪ Participant surveys ▪ Medicare claims ▪ AHA and TIEP databases	▪ Organizational
**Implementation** Participant’s use of intervention and the costs required	▪ Completion rate (# who completed study tasks/# enrolled) ▪ Unit cost/physician	▪ Participant surveys ▪ Applications ▪ Project progress reports	▪ Physician
**Maintenance** Extent to which use of the intervention occurs ≥ 6 months*	N/A	N/A	▪ Physician

*Medicare FFS* Medicare Fee-for-Service, *AHA* American Hospital Association, *TIEP* Trauma Information Exchange Program

##### Primary outcome (effectiveness)

Our primary outcome is the aggregated performance of physicians in the intervention and control groups when triaging trauma patients, assessed using Medicare claims data. Specifically, we will calculate the mean proportion of severely injured patients, evaluated by trial participants at a non-trauma center, who are *not* transferred to a level 1 or level 2 trauma center within 24 h (i.e., under-triage), as recommended by the clinical practice guidelines. We use under-triage as our primary outcome because it is an important measure of physician behavior, and because of its association with patient-centered outcomes, including mortality and return to work [22, 23]. We will define “severe injuries” using injury severity scores (ISS), with a cutoff > 15, consistent with the literature.

##### Secondary outcomes (effectiveness)

Secondary outcomes will include 30-day mortality and readmission (composite outcome), new-onset functional dependence (proportion of patients with 90-day pre-admission location at home with discharge to a skilled nursing or rehabilitation facility), and over-triage (patients with ISS < 15 who were transferred to a higher level of care). We use the 30-day composite outcome to increase statistical efficiency, balancing concerns of validity with feasibility (given the low base rate of individual outcomes) [24–26]. To assess harm, we will capture over-triage, which in theory could worsen outcomes for patients with other conditions at trauma centers by increasing treatment delays and reducing the availability of resources. Additionally, over-triage results in removal of patients from their community, without personal benefit.

##### Other RE-AIM outcomes 

We will also estimate the intervention’s reach (i.e., the number, proportion, and representativeness of the individuals willing to enroll in the trial), adoption (i.e., the absolute number, proportion, and representativeness of the settings), and implementation of the intervention (i.e., participants’ use and the costs required [e.g., honoraria]) [21].

#### Participant timeline

We summarize participant activities in Table 3. We will ask participants to use their intervention within 2 weeks of enrollment in the trial and quarterly during the subsequent 9 months (total time required: 3 h). They will complete web-based questionnaires assessing the intervention’s usability and reporting the fidelity of intervention delivery twice: immediately after completing the intervention the first time and then after their third booster dose at 9 months. These instruments will take less than 5 min to complete. We will also ask them to complete a web-based tool to measure physician behavior in trauma (SONAR) after the first use of the game. Participants in the usual education control will be asked to complete SONAR within 2 weeks of enrollment. Completing SONAR will take approximately 1 h.

**Table 3:**
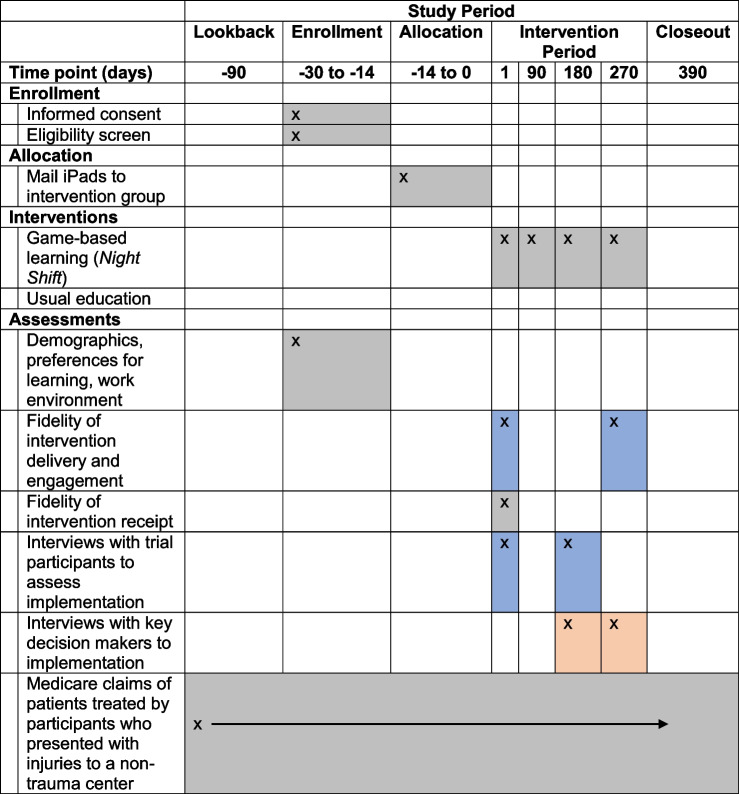
Schedule of enrollment, allocation, and assessment activities

Gray shading = both groups participate in activities

Blue shading = group allocated to game-based learning participates in activities

Orange shading = key decision makers recruited separately from trial participants

#### Sample size

Based on prior studies, we assume that ≥ 75% of enrolled physicians will encounter at least one eligible patient (i.e., enrolled in Medicare Fee-for-Service, severely injured, age ≥ 65 years), with a median number of patients per physician of 1–2. In this clustered randomization trial, physicians serve as the randomization unit. Based on data from a prior study, we assume an intraclass correlation coefficient (ICC) of 0.45, indicating the correlation in outcomes within the same physician group [27]. Under these assumptions, with 400 physicians per group (*N* = 800), we can detect between a 7.4–10% difference in under-triage between the intervention and control groups with 80% power and a significance level of 0.05, using a one-sided hypothesis test. We have chosen a one-sided hypothesis test to boost statistical efficiency and because we prioritize identification of a positive effect of the intervention. A negative effect would produce the same outcome as a null: the decision to pause further efforts to disseminate the intervention [28].

### Assignment of interventions: allocation and blinding

#### Sequence generation, concealment mechanism, and implementation

We will ask physicians to describe their personal characteristics at the time of enrollment and will include all minority (women, non-white) physicians who agree to participate, up to 50% of the targeted sample. Physicians will be randomized with an allocation ratio of 1:1, based on a randomization schema generated by our statistician (CCC) in STATA 17.0 (StataCorp, TX). Trial coordinators will link the randomization schema to the enrollment data and will assign participants to groups.

#### Who will be blinded

Although we cannot maintain blinding after allocation, our data analysts will not have access to that information.

#### Procedure for unblinding if needed

Since we cannot maintain blinding after allocation, we have not established a procedure for revealing a participant’s allocated intervention.

### Data collection and management

#### Plans for assessment and collection of outcomes

##### Physicians

Each physician who enrolls in the trial will complete a baseline questionnaire with items that capture age, gender, race/ethnicity, state in which they work, type of residency training (emergency medicine, internal medicine, family practice, other), type of fellowship training, trauma education (date of last ATLS certification, completion of trauma resuscitation module published by ABEM), professional characteristics (number of ED shifts completed each month, trauma center status of the hospital at which they work, assignment of an NPI), attitudes to game-based learning, and the amount of time and money they have spent on continuing medical education activities in the prior year. Participants will also complete questionnaires to assess the fidelity of intervention delivery and receipt. We summarize the methods that we plan to use to assess treatment fidelity in Table 4, using a checklist developed by Borelli et al. as part of the National Institutes of Heath’s (NIH’s) Behavior Change Commission’s effort to improve the replicability of health-related behavior change interventions [29–31].

**Table 4:** Treatment fidelity execution and assessment plan

Treatment fidelity strategies grouped by category	Plan for execution and assessment
**Treatment design** (defined as the extent to which the design of the trial ensures that the active ingredients of the intervention are operationalized, the measures capture theoretical constructs, and potentially threats to validity are identified)	
Treatment dose in the intervention condition • Length of contact • Number of contacts • Duration of contact over time • Content of treatment	4 contacts over a 9-month period: • 2 h of game play within 2 weeks of enrollment • 20 min of game play at 90 days, 180 days, and 270 days *Night Shift 2024* covers 10 trauma triage principles derived from the American College of Surgeons guidelines and a review of the literature (see Table 1 for additional details)
Treatment dose in the comparison condition • Length of contact • Number of contacts • Duration of contact over time • Content of treatment	Not applicable
Specification of provider credentials that are needed	Not applicable
Clear articulation of theoretical model on which the intervention is based	The dual process model of cognition (see eFigure 1 and Table 1)
Plan to identify potential confounders of trial results	Semi-structured interviews with a subset of trial participants (*n* = 20) and key decision makers (*n* = 80) conducted at month 1 and month 6 to understand contextual factors that modify adoption, implementation, and maintenance
Plan to address possible setbacks in delivery of the intervention (i.e., back systems or providers)	Loading of application to iOS Apple Store so that participants can access it independently
If more than one intervention is described, all are described equally well	Not applicable
**Training providers** (defined as strategies to standardize training between providers and monitoring provider skills over time)	
	Not applicable
**Delivery of treatment** (defined as treatment differentiation, treatment competency, and treatment adherence)	
Method of ensuring that the content and dose of intervention is delivered as specified	*Intervention*—game transmits details of game usage (cases completed, time spent) to a secure database whenever iPad linked to WiFi
Method to assess participants’ contact with the information	*Intervention*—information captured automatically from the application
Assessment of nonspecific treatment effects (i.e., competency or quality of delivery of intervention)	Not applicable
Use of treatment manual	Not applicable
Plan for the assessment of whether the active ingredients were delivered	We will categorize the number of trauma triage principles reviewed by trial participants (*n* = 10)
Plan for assessment of whether proscribed components were delivered (e.g., components that were unnecessary or unhelpful)	Not applicable
Plan to minimize contamination between conditions	Accessing the game requires a login id (i.e., an email address), which will allow us to determine if participants in the control group have played it
A priori specification of acceptable treatment fidelity (e.g., providers adhere to delivering > 80% of components)	We will define acceptable treatment fidelity as a minimum of 2 h of game play (*intervention*)
**Receipt of treatment** (defined as extent to which participants understand and have the ability to use the knowledge, skills, or recommendations communicated in the intervention)	
Assessment of the degree to which participants understood the intervention	Self-report after first round of game play (*intervention*)
Specification of strategies to improve participant comprehension of the intervention	Not applicable
Participant ability to perform the intervention skills is assessed during the intervention period	Use of SONAR (a tool designed to measure phy*S*ician behavi*O*r i*N* tr*A*uma t*R*iage) to quantify determinants of physician decision making after first round of game play (*intervention*) or completion of CME (*control*)
Multicultural factors considered in the development and delivery of the intervention (e.g., provided in native language; protocol is consistent with the values of the target group)	Playtesting of game with end users to refine content and game mechanics; stakeholder panel to provide advice about study protocol
**Enactment of treatment skills** (defined as the ability of participants to use behavioral skills and cognitive strategies in real-world settings)	
Participant performance of the intervention skills will be assessed in settings in which the intervention might be applied	Claims data to capture performance in real life during the study
A strategy will be used to assess performance of the intervention skills in settings in which the intervention might be applied	Indirect observation

##### Fidelity of intervention delivery

We define fidelity of intervention delivery as treatment adherence. The *Night Shift 2024* application collects data on the time that each player spends using the application, including the number of minutes, number of visits, and progress through the application. Each time the device connects to a wireless network, these data will be uploaded to a secure server hosted by the University of Pittsburgh. We will additionally ask participants in the intervention group to report game usage (e.g., time spent, most memorable case completed) and to complete the User Engagement Scale – Short Form (a validated 12-item instrument that measures esthetic appeal, attentional focus, perceived usability, and reward), after using *Night Shift* in the first month of the trial and again after the ninth month of the trial [32]. We provide the questionnaires in Additional file 1: Appendix.

##### Fidelity of intervention receipt

We define intervention receipt as evidence that participants understand and can use the skills or knowledge learned during the intervention and will evaluate it in three ways. First, we will ask participants (intervention and control) to describe their understanding of the central principle of trauma triage on the first post-enrollment survey. Second, we will ask them to complete an online tool that assesses physician behavior in trauma triage (SONAR). SONAR is a 2D web-based serious game, during which physicians must triage 30 trauma patients (15 with severe injuries; 15 with minor injuries). The game evaluates decisions using the guidelines published by the American College of Surgeons – Committee on Trauma and produces two sets of metrics: compliance with guidelines (e.g., under-triage) and signal detection parameters. Signal detection theory came to prominence during World War II and allows inferences about the sources of non-compliance with clinical practice guidelines by parsing the influence of perceptual sensitivity (the ability to distinguish between minimally and severely injured patients) and decisional thresholds (preferences to err on the side of under- or over-triage) on decisions [33]. Third, we will interview a subset of physicians in the game arm (*n* = 20) at 1 and 6 months after enrollment, to learn about their experiences with the game and contextual modifiers to adoption, implementation (anticipated/actual), and maintenance (anticipated/actual) of guideline-concordant trauma triage. We will supplement this data with interviews of a national sample of other key decision-makers (i.e., patients, ED directors at non-trauma centers, trauma directors at level I/II trauma centers, first responders [*n* = 80]). We will focus on barriers and facilitators of implementation of clinical practice guidelines in trauma triage (anticipated [month 1] and actual [month 6]) using an interview guide developed using the Consolidated Framework for Implementation Research (CFIR) [21]. We include the interview guides in Additional file 1: Appendix.

##### Hospitals

We will obtain information about the organizational characteristics of each hospital at which physicians work using the 2022 Centers for Medicare and Medicaid Services (CMS) Healthcare Cost Report Information System (HCRIS). HCRIS contains facility-level characteristics of all non-federal hospitals, including geographic location (state, region), participation in a hospital network, total bed count, intensive care unit (ICU) bed count, ownership, and teaching status. Since HCRIS does not contain data on the trauma center status of hospitals, we will link HCRIS to the Trauma Information Exchange Program (TIEP) to identify the trauma center designation for each hospital in 2023.

##### Patients

To construct the dataset that we will use to analyze the performance of physicians, we will obtain Inpatient, Outpatient, and Professional Claims filed with Medicare Fee-for-Service (FFS) and Advantage for beneficiaries older than 65 years old with International Classification of Diseases, Tenth Revision (ICD10) codes associated with injuries in 2024. We will also obtain claims for the quarters (Qs) flanking 2024 (i.e., Q4 2023, Q1 2025) to enable us to identify preceding and follow-up care after injuries. Data elements abstracted directly from the claims will include patient demographics, the hospital identifier, date of admission/discharge, ICD10 diagnosis/procedure codes, disposition status (e.g., home, nursing home), and vital status (date of death). We will map ICD10 diagnosis codes to abbreviated injury scores (AIS) using a well-validated program (ICDPIC) and will calculate injury severity scores (ISS). We will also estimate the presence or absence of serious illness and organ failure using validated algorithms [34, 35]. Finally, we will estimate functional status pre-injury by conducting a 90-day lookback from the date of admission to identify claims filed at skilled nursing or rehabilitation facilities.

We will identify patients treated by trial physicians by linking the names of trial participants to NPIs and searching for claims filed by those physicians in the Inpatient, Outpatient, and Professional Claims files. We will then construct episodes of care for each patient by linking Outpatient and Inpatient Standard Analytic files to identify visits to acute care, non-federal hospitals. We will order claims by day and classify visits that occur within 1 day of each other as part of a single episode of care. For episodes with multiple claims from the same day, we will order the claims under the assumption that patients will move from non-trauma centers to trauma centers, and from low-volume hospitals to high-volume hospitals.

#### Data management

Data sources include consent forms collected electronically, survey data collected electronically, audio files and transcripts of interviews, and claims files purchased from CMS. All data will be stored on a secure server at the University of Pittsburgh. Data integrity checks will be conducted periodically every 6 to 12 months by the principal investigator and the security team for the University of Pittsburgh School of Medicine Information Technology department. Additional processes to promote data quality will include range checks for data values and analysis by two different statisticians.

#### Confidentiality

We will create a linkage file that connects personal data with anonymized identifiers and then will use de-identified data for all analyses. This file will be encrypted and stored on our secure server, and only the study team will have access to it.

### Plans for collection, laboratory evaluation, and storage of biological specimens for genetic or molecular analysis in this trial/future use

Given the nature of the trial, we have not developed any plans for biological specimens.

### Statistical methods

We will use summary statistics to describe physician, hospital, and patient-level variables and will describe patterns of missingness to identify variables with non-random and high (≥ 10%) missingness. We will also calculate measures of reach (proportion of those received [and opened] an email invitation who completed a screening form and the demographics of those who responded compared to those who meet eligibility criteria), adoption (number, proportion, and characteristics of hospitals staffed by trial physicians compared to other non-trauma centers), and implementation (the proportion of those who enrolled who completed study tasks, the unit costs per physician).

#### Fidelity of intervention delivery and receipt

We will summarize the proportion of time that physicians spend using their intervention, and the proportion who provide a complete answer to the attention check question. We will also summarize responses to the question about the principle that guides trauma triage. Finally, we will estimate measures of compliance with guidelines and signal detection measures of the determinants of those decisions from SONAR and will compare them between groups using analysis of variance (ANOVA). Differences between groups in parameters of compliance or signal detection theory would suggest differential receipt of learning principles embedded in the different applications.

#### Statistical methods for primary and secondary outcomes

Our hypotheses are related to the effectiveness of the intervention and are listed in Table 5.

**Table 5:** List of hypotheses to be tested

Type of hypothesis	Specification of the hypothesis
Primary (behavioral analysis)	Physicians in the game-based training group will under-triage a smaller proportion of patients than those in the usual education group
Secondary (outcome analysis)	Physicians in the game-based training group will have fewer patient outcomes (i.e., 30-day mortality and readmissions, loss of functional independence) than those in the usual education
Secondary (outcome analysis)	Physicians in the game-based training group will over-triage a similar proportion of minimally injured patients compared to those in the usual education group
Secondary (mediation)	Under-triage will mediate the effect of the intervention on patient outcomes
Exploratory (heterogeneity of treatment effect)	[1] The intervention will have the same effect on males as on female trial participants [2] The intervention will have the same effect on White as on non-White trial participants [3] The intervention will have a greater effect on trial participants who express positive attitudes to game-based learning before enrollment compared to those who do not have positive attitudes [4] Trial participants in the game-based training group who use *Night Shift 2024* for greater than 2 h will under-triage a smaller proportion of severely injured patients than those who use the game for less than 2 h [5] The proportion of patients under-triaged by trial participants will be lower immediately after exposure to the intervention (i.e., in the first 30 days) compared to late post-exposure period (i.e., 30–89 days) [6] Trial participants with greater parameters of compliance on SONAR (the experimental tool to measure determinants of physicians’ decision making) will under-triage a smaller proportion of severely injured patients than those with lower parameters of compliance

##### Primary and sensitivity analyses

To evaluate physician performance, we will create a cohort of patients as described above in the “Data collection and management” section and will restrict analysis to patients with a severe injury (ISS ≥ 15), to the first episode of care for each patient (since we cannot determine if subsequent episodes reflect follow-up care or new injuries), and to patients treated for their first episode of care in 2024 (i.e., after rollout of the intervention). We will exclude patients who died on the day of admission (as this could reflect either an error in triage decision making or an assessment of clinical instability that precluded transfer) and patients who were discharged from the ED (as this could reflect either an error in triage decision making or an error in the coding of hospital records). We will also exclude patients who presented initially to a level I–IV trauma center, since the guidelines for triage focus on under-triage at non-trauma centers. Finally, we will classify patients with ISS ≥ 15 as triaged appropriately (if they were transferred to a higher level of care within 24 h of presenting to the hospital) or under-triaged (if they were admitted to the non-trauma center).

To estimate the effect of the intervention on physician behavior, we will calculate under-triage (1-proportion of patients with severe injuries successfully transferred to trauma centers) for physicians in each arm of the trial. We plan to use an intention-to-treat approach, including all randomized physicians (regardless of their degree of participation in the study) if we allocated them to treatment and if they filed Medicare claims. We will compare differences in post-intervention behavior between groups using a generalized linear mixed model (GLMM) predicting under-triage at the level of the patient, with binomial error distribution and log or logit link function depending on outcome is rare or not, and adjusting for baseline covariates (hospital-level [bedsize, teaching status, participation in a healthcare system, resource availability], physician-level [demographics, type of board certification, ATLS certification, patient load], and patient-level ones [demographics, injury severity scores, organ failure]). Fixed effects in the model will include intervention groups, intervention period, and their interactions. Random effects will include hospital- and physician-level random intercepts. If the model does not converge (due to the scarcity of cases within hospitals), we will remove the hospital-level random effect preferentially.

In sensitivity analyses, we will test if the effect is modified by candidate moderators (e.g., physician experience) by testing the interaction between the moderator and the group assignment. Finally, we will test alternative definitions of under-triage (including the more restrictive categorization of any patient not transferred to a level I/II trauma center as under-triaged).

##### Interim analyses

We are not planning any interim analyses.

##### Methods for additional analyses

To test the effect of the interventions on patient-centered outcomes, we will repeat the GLMM analyses using different dependent variables: over-triage, composite 30-day patient outcome, and functional dependence. We will build regression models in which we will calculate the direct, indirect, and total effects of the interventions on patient-centered outcomes, testing the mediation exerted by under-triage. Finally, we will explore the heterogeneity of the treatment effect by evaluating the effect of the intervention on different cohorts of participants: women v. men; white v. non-white; those with positive v. negative attitudes to game-based learning. Additional heterogeneity of treatment effect analyses include a test of the dose effect of the intervention, the durability of the treatment effect, and the impact of intervention receipt.

##### Methods in analysis to handle protocol non-adherence and any statistical methods to handle missing data

We plan to use an intention-to-treat approach to evaluate the effect of the video game on physician triage decision making. The GLMM assumes that missing data is missing at random (MAR). If that assumption holds, and some patient covariates exhibit a higher percentage of missingness, we will carry out multiple imputations before fitting the GLMM model. This is to ensure that the statistical power remains not less than 80%. However, in secondary analyses (as described above), we will also test a per-protocol approach, categorizing physicians based on their completion of assigned study tasks. We also plan sensitivity analyses to handle non-random missingness using a joint modeling approach.

##### Plans to give access to the full protocol, participant-level data, and statistical code

The protocol, primary data (i.e., physician-level data), summary data, and meta-data (e.g., documentation, protocols used to clean and to manage the data) will be uploaded to the open access Inter-university Consortium for Political and Social Research (open ICPSR) repository at the conclusion of the trial. Data use agreements for Medicare claims typically preclude sharing of data, so patient-level files cannot be distributed. However, we will make available the processes that we use to create administrative linkages between trial data and the claims files.

### Oversight and monitoring

#### Composition of the trial team and stakeholder advisory committee

The trial team will comprise of the investigators, coordinators, and staff members. They will meet monthly initially to establish and adjust the study protocol as necessary. Subsequently, they will meet quarterly to discuss study progress and interim results, as well as respond to any issues that have arisen. They will receive input from a stakeholder advisory committee, comprised of a diverse group of local and national leaders in trauma care (*n* = 9). These stakeholders vary in their demographics, training, experience, and work environment. We will convene the panel every 6 months via video-conference to obtain feedback on all phases of the study, from startup to close-out.

#### Composition of the data monitoring committee, its role and reporting structure

The University of Pittsburgh Human Research Protection Office (HRPO) has reviewed our protocol and provided approval (STUDY23070156). The funding agency (the National Institute on Aging [NIA]) will convene an independent Data and Safety Monitoring Board (DSMB) who will also review and approve the protocol and the data monitoring plan. The DSMB will meet before the start of the trial and then every 9–12 months until analysis is completed. We do not plan any interim analyses and therefore have not included any stopping guidelines. We have registered the trial on ClinicalTrials.gov.

#### Adverse event reporting and harms

The primary investigator (PI) will ask participants to communicate any adverse events or unintended effects of participation via email, which she will relay to the review boards. Physicians may opt to withdraw from the trial at any point, at which time we will exclude all self-reported data from analysis.

#### Frequency and plans for auditing trial conduct

There is no set frequency for audits of trial processes and protocols.

#### Plans for communicating important protocol amendments to relevant parties

All protocol amendments will be communicated to the DSMB, to the trial sponsor, and to the HRPO at the University of Pittsburgh.

#### Dissemination plans

Results from the study will be reported to the public through manuscripts and oral presentations at national meetings. All investigators and stakeholders will have the opportunity to serve as authors on manuscripts generated from this research as long as they have made a substantial contribution, have reviewed it for content, provide approval of the final manuscript, and agree to be accountable for the accuracy and integrity of the work.

## Discussion

This protocol paper outlines a clinical trial to test the effectiveness of a video game at increasing the implementation of clinical practice guidelines in trauma triage. Strengths of the trial include the addressing of the national priority of maintaining the health and independent living of older adults, the testing of an intervention explicitly grounded in theory and proven efficacious in the laboratory, and a pragmatic mixed-method process evaluation that will allow interpretation of both negative as well as positive results.

We confronted several design challenges during the development of the study. First, we debated the optimal comparator for our intervention. ATLS, a 2-day textbook based educational program sponsored by the American College of Surgeons, represents the gold-standard for continuing medical education in trauma triage [18]. Participants attend lectures covering core topics, practice unfamiliar skills (e.g., chest tube insertion), and demonstrate knowledge acquisition by completing a pre- and post-test. The American College of Surgeons recommends that physicians take the course quadrennially, and provides certification of competence to credentialing organizations (e.g., hospitals). The logistics and cost required to enroll trial participants in the course made this option infeasible. Additionally, we did not design *Night Shift 2024* to replace ATLS. Instead, it ideally serves as an adjunct, facilitating the type of distributed training that encourages the retention and use of best-practice decision making principles. It offers an alternative to the continuing medical education that physicians ordinarily complete to satisfy annual requirements of state medical boards. We therefore selected usual education as our comparator. We considered but rejected the idea of using two comparators (usual education and enhanced usual education) in the interests of statistical efficiency for testing the intervention.

A second design challenge involved the decision of how best to assess intervention receipt (i.e., do trial participants understand the information provided in the intervention), a core component of treatment fidelity. The NIH’s Behavior Change Commission recommends using pre- and post-test measures of process, skills, and knowledge for this purpose [29]. However, measurement of physician judgment before-and-after exposure to the intervention (a within-subject analysis) requires that participants complete SONAR twice, increasing respondent burden and the likelihood of attrition. We therefore decided to use a between-subject analysis, comparing the judgment of physicians exposed to game-based and text-based learning.

A third design challenge was determining the optimal dose of the triage video game that participants would receive. In prior laboratory studies, we found that physicians exposed to 2 h of game-based learning experienced a greater effect than those who completed 1 h [16, 17]. Pedagogical research shows the value of distributed exposure to educational interventions, to allow the transference of information from the working to the long-term memory [36]. However, the longer the intervention delivery period, the greater the risk of attrition. We therefore compromised by designating the dose as 2 h of game play immediately after enrollment, followed by 20-min booster sessions quarterly. As mentioned, we propose secondary analyses relating the dose (total number of minutes played and number of sessions) to effectiveness.

The study has several limitations. One primary concern is the reliance on claims to assess the effectiveness of the intervention. For example, the identification of the patient cohort requires the use of ISS derived from ICD10 codes, which have less sensitivity and specificity than the gold standard of scores calculated by trauma registrars after chart review. However, the method of using ICD10 codes to measure injury severity is well-validated (kappa 0.76–0.92), offers a reasonable proxy for the full clinical record, and makes the current project feasible [37–39]. The use of Medicare claims also allows the recruitment of a national sample of physicians, increasing the generalizability of observations. Another potential limitation of the study arises from the use of incentives to recruit and to retain physician participants, which introduces the potential for selection bias. However, we believe that this approach is consistent with the NIA’s recommendation to prioritize the fidelity of intervention delivery (internal validity) during initial effectiveness testing over the representativeness of the sample (external validity) [28]. Finally, the study intervenes on only one determinant of non-compliance with clinical practice guidelines (physician heuristics), even though multiple variables contribute to the problem (e.g., structural constraints, capacity issues). However, physicians represent the largest source of variation in triage practices; interventions that effectively modify their behavior have the potential to offer novel solutions to the refractory problem of poorly calibrated heuristics in medicine.

Advances in technology hold the potential to transform the delivery of behavioral and social science interventions. They improve treatment fidelity and can increase the acceptability of distributed delivery, thus improving behavioral maintenance. We have developed one such behavioral intervention to recalibrate physician heuristics in trauma triage and plan to test its efficacy. We intend that results of this trial will contribute to the literature on physician quality improvement and the efficacy of video games as behavioral interventions.

Trial status: Not yet recruiting.

Anticipated start date for recruitment: November 27, 2023.

Anticipated completion date for recruitment: February 15, 2024.

Protocol version: 2

Date: 4 October 2023.

### Supplementary Information

**Additional file 1: Appendix. eFigure 1.** Trial schematic. **eFigure 2.** Conceptual model of intervention.

## Data Availability

*Night Shift 2024* is available for download on the iOS Apple Store at https://apps.apple.com/us/app/night-shift-2024/id6448066837. *SONAR* will be available for use at https://howdodoctorsthink.study.ccm.pitt.edu/.

## Abbreviations

ABEM: American Board of Emergency Medicine
ANOVA: Analysis of variance
ATLS: Advanced Trauma Life Support
CFIR: Consolidated Framework for Implementation Research
CMS: Centers for Medicare and Medicaid Services
DSMB: Data Safety Monitoring Board
ED: Emergency department
FFS: Fee-For-Service
GLMM: Generalized linear mixed model
HCRIS: Healthcare Cost Report Information System
HRPO: Human Research Protection Office
ICC: Intraclass Correlation Coefficient
ICD10: International Classification of Diseases, Tenth Revision
ICPSR: Inter-university Consortium for Political and Social Research
ICU: Intensive care unit
ISS: Injury severity scores
MAR: Missing at random
NIA: National Institute on Aging
NPI: National Provider Identifier
Q: Quarter
PI: Primary investigator
RE-AIM: Reach, Effectiveness, Adoption, Implementation, Maintenance
TIEP: Trauma Information Exchange Program
US: United States
