# Supplementary material for: Using a theory-based, customized video game as an educational tool to improve physicians’ trauma triage decisions: study protocol for a randomized cluster trial
Source: Trials. 2024 Feb 16;25:127. doi: 10.1186/s13063-024-07961-w (PMC10870723; doi:10.1186/s13063-024-07961-w)
Supplement: Supplementary file 1 — Additional file 1: Appendix. eFigure 1. Trial schematic. eFigure 2. Conceptual model of intervention. [file 13063_2024_7961_MOESM1_ESM.docx]

# Appendix

Using a theory-based, customized video game as an educational tool to improve physicians' trauma triage decisions: a study protocol for a randomized clinical trial

Table of Contents

[Appendix 1](#_Toc147246182)

[Consent form 3](#_Toc147246183)

[Instruments 5](#_Toc147246184)

[Screening questionnaire 5](#_Toc147246185)

[Instrument to assess fidelity of intervention delivery (month 1) - intervention group 6](#_Toc147246186)

[Instrument to assess fidelity of intervention receipt (month 1) - intervention and control groups 6](#_Toc147246187)

[Instrument to assess fidelity of intervention delivery (month 10) - intervention group 7](#_Toc147246188)

[Interview guides 8](#_Toc147246189)

[Trial participants (month 1 and month 6) 8](#_Toc147246190)

[Trauma directors 10](#_Toc147246191)

[Ed directors 12](#_Toc147246192)

[Paramedics 15](#_Toc147246193)

[Patients 17](#_Toc147246194)

[Surrogates 19](#_Toc147246195)

[Efigure legends 21](#_Toc147246196)

## Consent form

Thank you for your interest in this NIH-funded research study. My name is xxxx and I am a researcher at the University of Pittsburgh – School of Medicine. The purpose of this study is to test the effect of a video game on the implementation of clinical practice guidelines in trauma triage. Specifically, we are interested in whether video games have a greater effect on physician behavior than more conventional continuing medical education, like reading journal articles.

As part of this study, we will be randomizing 800 physicians to one of two arms: the intervention or enhanced usual care (control). Physicians in the intervention group will be asked to play a video game (*Night Shift*) for two hours within two weeks of enrollment, and then return to the game for three twenty minute booster sessions at 3 month intervals. They will be asked to report on their experience with the game after the initial two hour session, and again after the third booster session. They will also be asked to complete a web-based tool that assesses decision making, after playing the game for the first time. Participants in the control group will be asked to complete two hours of continuing medical education of their choice and to submit proof of completion to the study team. They will also be asked to complete the web-based tool that assesses decision making. Completing study tasks will take either three or four hours over nine months, and can be completed at the convenience of the participant. We will provide all trial participants with an honorarium of $100/hour spent on study tasks. As per IRS guidelines, all compensation is taxable income to the participant regardless of the amount. If a participant receives $600 or more in a calendar year form one organization, that organization is required by law to file a Form 1099-Miscellaneous with the IRS and provide a copy to the taxpayer. Individuals who do not provide a social security number may still participate in the research, but the IRS requires that 28% of the payment be sent by the institution to the IRS for 'backup withholding;' thus you would only receive 72% of the expected payment.

There will be no direct benefit of participation to you. We will be linking your responses to claims data to identify how you make decisions for patients in the Emergency Department, and the effect of the intervention on those decisions. The primary risk would be a breach of confidentiality, which might damage your reputation if your description of how you manage patients is judged in a negative fashion. To prevent this from happening, the identifiers will be stored separately, and your responses will be coded and stored on a secure server maintained by the Data Center in the Department of Critical Care Medicine.

The linkage file that ties your name to the identifier will be kept on a secure server behind the University of Pittsburgh firewall. The research team, the University of Pittsburgh Office of Research Conduct and Compliance, and personnel from the NIH, will have access to the research records. A description of this clinical trial will be available on http:///www.ClinicalTrials.gov, as required by U.S. Law. This Web site will not include information that can identify you. At most, the Web site will include a summary of the results. You can search the web site at any time. Finally, your research data may be shared with investigators conducting other research; however, this information will be shared in a de-identified manner (without identifiers).

Obviously, your participation in this study is completely voluntary (and much appreciated). You may withdraw at any time. Should you choose to withdraw, all date will continue to be used up to the point of withdrawal unless you request that we destroy it. There is no penalty for refusing to participate or withdrawing. A description of the clinical trial will be available on http://www.clinicaltrials.gov, as required by US Law. This website will not include information that can identify you. At most the website will include a summary of the results. You can search this website at any time. Any questions or concerns should be directed to me as the principal investigator in this study: xxxx. My cell phone number where I can be reached is xxx-xxx-xxxx. If you have any concerns about the study or your rights as a participant, you can contact the University of Pittsburgh human subject protection advocate phone line (1-866-212-2668).

If you are willing to participate, please fill out the following items below which will be used as an electronic signature:

1. Full name
2. Birthdate
3. Name of your high school
4. Are you willing to participate in this study (yes/no)
5. Do you provide your permission to be audio/video recorded for the purposes of this study (yes/no).

## Instruments

### Screening questionnaire

| **Question** | | **Response options** |
| --- | --- | --- |
| How old are you? | | Years (range 25-80) |
| What is your gender? | | Male, female, prefer not to say |
| What is your race? | | Alaska Native, Native American, Hawaiian, or Pacific Islander  Asian  Black and African American  White |
| What is your ethnicity? | | Hispanic, not hispanic, prefer not to say |
| In what region of the country do you work? | | Northeast, Southeast, Midwest, Southwest, Northwest |
| Are you board-certified in a medical specialty? | | Yes/No |
|  | If yes, which board? | Emergency Medicine  Family Practice  Internal Medicine  General Surgery  Other |
| Have you completed fellowship training? | | Yes/No |
|  | If yes, which type of fellowship? | Free text |
| Have you completed the ATLS course? | | Yes/No |
|  | If yes, when? | <1 year ago  ≥1-4 years ago  >4 years ago |
| Have you completed the trauma resuscitation module published by ABEM? | | Yes/No |
|  | If yes, when? | <1 year ago  ≥1 year ago |
| How many shifts do you work in the ED each month on average? | | 0-31 |
| What is the trauma center designation of the hospital at which you practice? | | 0 (non-trauma center)  5, 4, 3, 2, 1 |
| If you practice at more than one hospital, does any of the hospitals have a trauma center designation | | Yes/No |
| Do you play video games for fun? | | Yes/No |
| How many hours would you estimate that you spend on continuing medical education activities in 2023? | | <10 hours; 10-25 hours; 26-50 hours |
| How much would you estimate that you spend on continuing medical education activities in 2023? | | free text |

### Instrument to assess fidelity of intervention delivery (month 1) - intervention group

| **Question** | | **Response options** |
| --- | --- | --- |
| How long did you spend playing Night Shift? | | 0-30 minutes, 30-60 minutes, 60-90 minutes, 90-120 minutes, >120 minutes |
| What case do you remember best? | | Free text |
|  | What made the case memorable? | Free text |
| How would you evaluate your experience? | |  |
| I felt interested in this experience | | Strongly disagree; disagree; neutral; agree; strongly agree |
| My experience was rewarding | | Strongly disagree; disagree; neutral; agree; strongly agree |
| Using *Night Shift* was worthwhile | | Strongly disagree; disagree; neutral; agree; strongly agree |
| *Night Shift* appealed to my senses | | Strongly disagree; disagree; neutral; agree; strongly agree |
| *Night Shift* was aesthetically appealing | | Strongly disagree; disagree; neutral; agree; strongly agree |
| *Night Shift* was attractive | | Strongly disagree; disagree; neutral; agree; strongly agree |
| Using *Night Shift* was taxing | | Strongly disagree; disagree; neutral; agree; strongly agree |
| I found *Night Shift* confusing to use | | Strongly disagree; disagree; neutral; agree; strongly agree |
| I felt frustrated while using *Night Shift* | | Strongly disagree; disagree; neutral; agree; strongly agree |
| I was absorbed in the experience | | Strongly disagree; disagree; neutral; agree; strongly agree |
| The time slipped away | | Strongly disagree; disagree; neutral; agree; strongly agree |
| I lost myself in the experience | | Strongly disagree; disagree; neutral; agree; strongly agree |

### Instrument to assess fidelity of intervention receipt (month 1) - intervention and control groups

| How would you describe the guiding principle of trauma triage? | Free text |
| --- | --- |

### Instrument to assess fidelity of intervention delivery (month 10) - intervention group

| **Question** | **Response options** |
| --- | --- |
| How long did you spend playing Night Shift in total? | 0-30 minutes, 30-60 minutes, 60-90 minutes, 90-120 minutes, >120 minutes |
| How would you evaluate your experience with the intervention as a whole? |  |
| I felt interested in this experience | Strongly disagree; disagree; neutral; agree; strongly agree |
| My experience was rewarding | Strongly disagree; disagree; neutral; agree; strongly agree |
| Using *Night Shift* was worthwhile | Strongly disagree; disagree; neutral; agree; strongly agree |
| *Night Shift* appealed to my senses | Strongly disagree; disagree; neutral; agree; strongly agree |
| *Night Shift* was aesthetically appealing | Strongly disagree; disagree; neutral; agree; strongly agree |
| *Night Shift* was attractive | Strongly disagree; disagree; neutral; agree; strongly agree |
| Using *Night Shift* was taxing | Strongly disagree; disagree; neutral; agree; strongly agree |
| I found *Night Shift* confusing to use | Strongly disagree; disagree; neutral; agree; strongly agree |
| I felt frustrated while using *Night Shift* | Strongly disagree; disagree; neutral; agree; strongly agree |
| I was absorbed in the experience | Strongly disagree; disagree; neutral; agree; strongly agree |
| The time slipped away | Strongly disagree; disagree; neutral; agree; strongly agree |
| I lost myself in the experience | Strongly disagree; disagree; neutral; agree; strongly agree |

## Interview Guides

### Trial Participants (Month 1 and Month 6)

My name is _____and I am a researcher from the department of Critical Care Medicine at the University of Pittsburgh. This study is being conducted by Principal Investigator, Dr. Deepika Mohan, Associate Professor of Critical Care Medicine and Surgery, and colleagues to better understand the environment in which you make triage decisions.

I will be asking you several questions about your experience with trauma triage. There are no right answers to these questions. Your responses will be completely confidential and will in no way affect your employment. Although participating in this interview has no direct benefits to you, your responses may help to improve future educational experiences.

We can stop the interview at any point if you feel uncomfortable or do not wish to continue. Also, you do not have to answer any questions that make you feel uncomfortable.

**Do you have any questions at this time? Do I have your permission to audio record our conversation?**

**[Begin recording. State the date, time, and participant identification number]**

**NB: Skip highlighted questions if time is short**

**First, I would like to learn your thoughts and opinions on a trauma case.**

1. Let’s imagine an 80-year-old has fallen and has multiple rib fractures. She’s saturating reasonably well but requires additional O2. What would you do with that person? (keep, transfer, discharge) What would you consider in making your decision?
2. Do you have a protocol for management of trauma patients in your ED?
   1. *(If yes)* What do you think of the protocol?
   2. How would you typically find out if there were new policies that were implemented at your hospital related to trauma triage? *Do you meet formally or informally to discuss trauma triage policies?
   3. *(If no*) Are there norms or expectations at your hospital related to trauma triage? (probe: from leadership, consultants, ED team, financial)
3. Who is involved in trauma triage decisions?
   1. What is the team workflow/workload infrastructure to make triage decisions?
4. How is your ED physically organized to manage trauma patients? [separate space for trauma patients?]
   1. How does the physical space of the ED support trauma triage? Hinder?
5. Who would you go to if you are unsure about a trauma triage decision?
   1. Who do you go to if you disagree with a triage decision?
6. Do you know if your hospital is required to report their trauma triage performance to anyone? [number of patients seen, number treated, number transferred]

**Next, I would like to hear about external factors that might impact trauma triage.**

1. What infrastructure is in place to get trauma patients to a higher level of care? [beds, transportation, accepting patient, insurance, geography, system affiliation, partnerships, logistics of transfer, receiving characteristics]
2. What are the typical patient and surrogate preferences in relation to being transferred for trauma?
   1. How frequently do you have a conversation with patients about their transfer preferences?
   2. To what extent are the needs of patients and surrogates considered when deciding how to triage patients?

**My last set of questions are about your experience and opinions of the Night Shift game to learn about trauma triage.**

1. Tell me about your experience with Nightshift.
2. How appropriate was the game play/session for you?
   1. How relevant did you find the demonstration of behavior(s) in the game?
3. How valuable were the game play sessions? (The information in the game)
   1. How would you rate Nightshift in comparison to other continuing medical education?
   2. How much did you identify with "Andy"?/Could you empathize with Andy as he went through the scenarios?
   3. How realistic did you find the consequences within the game?
   4. Could you empathize with Andy as he received consequences of his behavior?
4. Have you been able to use any of the information provided in game play?
   1. Have you experienced any challenges in implementing the information provided?

### Trauma Directors

My name is _____and I am a researcher from the department of Critical Care Medicine at the University of Pittsburgh. This study is being conducted by Principal Investigator, Dr. Deepika Mohan, Associate Professor of Critical Care Medicine and Surgery, and colleagues to better understand the environment in which you make triage decisions.

I will be asking you several questions about your experience with trauma triage. There are no right answers to these questions. Your responses will be completely confidential and will in no way affect your employment. Although participating in this interview has no direct benefits to you, your responses may help to improve future educational experiences.

We can stop the interview at any point if you feel uncomfortable or do not wish to continue. Also, you do not have to answer any questions that make you feel uncomfortable.

**Do you have any questions at this time? Do I have your permission to audio record our conversation?**

**[Begin recording. State the date, time, and participant identification number]**

**NB: skip highlighted questions if time is short**

**First, I would like to learn your thoughts and opinions on guidelines for trauma triage.**

1. Tell me about the guidelines for the transfer of trauma patients in your region.
   1. How have the guidelines been adapted so they work in your region?
   2. What evidence supports the guidelines? (are you aware of any studies or other supporting evidence?)
   3. In your experience, how effective are the guidelines? (do you think the guidelines change patient outcomes?)
   4. How do you find out new information about updates to trauma triage best practices? (journals, colleagues, etc)
2. What do you do to promote the guidelines? (trau*ma centers do outreach)*
   1. How easy or hard is it to promote the guidelines?

**(inner setting) Now, I would like to ask about the infrastructure of your ED related to trauma triage.**

1. What infrastructure is in place to transfer trauma patients? [beds, transportation, accepting patient, insurance, geography, system affiliation, partnerships, logistics of transfer, receiving characteristics]
2. What kind of capacity issues exist for you? [How easy or hard is it to take trauma patient influx]
   1. How do you weigh clinical needs with capacity constraints when making decisions about accepting trauma transfers?
3. Are there norms or expectations at your hospital related to trauma triage? (can probe from up to CMO - person who is responsible for decisions about capacity and patient flow - or down to ED director)
   1. Are there any challenges to upholding these expectations?
   2. What financial incentives are there to accept transferred patients? To not accept patients?

**(outer setting) Next, I’d like to ask about factors outside of your organization that may impact trauma triage.**

1. What are your relationships with the outlying hospitals that refer to you? What structures are in place to facilitate the transfer process?
2. Do you internally review cases of trauma triage?
   1. What is the process by which you review cases of trauma triage? What kind of feedback do you provide in following up with your referral centers? How frequently does that feedback occur? What is the medium by which it occurs?
   2. What kind of feedback or communication happens from referral center to you? How often does that happen? How does that occur?
3. How competitive is your market in trauma triage? How does that influence the way you have structured trauma triage?

**To wrap up, I’d like to ask you a final question about patient preferences.**

1. To what extent do you think the preferences of patients and surrogates considered when deciding to implement trauma triage guidelines? [Outer Setting; Patient Needs & Resources]

### ED Directors

My name is _____and I am a researcher from the department of Critical Care Medicine at the University of Pittsburgh. This study is being conducted by Principal Investigator, Dr. Deepika Mohan, Associate Professor of Critical Care Medicine and Surgery, and colleagues to better understand the environment in which you make triage decisions.

I will be asking you several questions about your experience with trauma triage. There are no right answers to these questions. Your responses will be completely confidential and will in no way affect your employment. Although participating in this interview has no direct benefits to you, your responses may help to improve future educational experiences.

We can stop the interview at any point if you feel uncomfortable or do not wish to continue. Also, you do not have to answer any questions that make you feel uncomfortable.

**Do you have any questions at this time? Do I have your permission to audio record our conversation?**

**[Begin recording. State the date, time, and participant identification number]**

**NB: skip highlighted questions if time is short**

**First, I would like to learn your thoughts and opinions on guidelines for trauma triage.**

1. Tell me about the guidelines for the transfer of trauma patients in your region.
   1. How have the guidelines been adapted so they work in your region?
   2. What evidence supports the guidelines? (are you aware of any studies or other supporting evidence?)
   3. In your experience, how effective are the guidelines? (do you think the guidelines change patient outcomes?)
   4. How do you find out new information about updates to trauma triage best practices? (journals, colleagues, etc)
2. How are trauma triage guidelines similar to or different from other protocols that exist such as stroke in your region?

**(inner setting) Now, I would like to ask about the infrastructure of your ED related to trauma triage.**

1. What infrastructure is in place to transfer trauma patients? [beds, transportation, accepting patient, insurance, geography, system affiliation, partnerships, logistics of transfer, receiving characteristics]
2. What kind of capacity issues exist for you for transfer? [How easy or hard is it to send trauma patients]
   1. How do you weigh clinical needs with capacity constraints when making decisions about transferring trauma patients?
3. Are there norms or expectations at your hospital related to trauma triage? (can probe up to hospital leadership - person who is responsible for decisions about capacity and patient flow - or down to ED teams)
   1. Are there any challenges to upholding these expectations?
   2. What do you do to promote those expectations?
      1. How easy or hard is it to promote those expectations?
   3. What are financial considerations related to the decision whether or not to transfer trauma patients?

**(outer setting) Next, I’d like to ask about factors outside of your organization that may impact trauma triage.**

1. What are your relationships like with the hospitals that you refer trauma patients to?
2. What kind of feedback or communication happens internally to review cases of trauma triage?
   1. What kind of feedback do you provide in following up with your trauma centers? How frequently does that feedback occur? What is the medium by which it occurs?
   2. What kind of feedback or communication happens from the trauma center to you? How often does that happen?

**Next, I’d like to ask you about patient preferences.**

1. What are the typical patient and surrogate preferences in relation to being transferred for trauma?
2. What are some of the circumstances under which patients and surrogates would prefer not to be transferred? (What do you do in those circumstances?)
3. To what extent are the preferences of patients and surrogates considered when deciding to implement trauma triage guidelines?

**I have a couple of final questions to wrap up.**

1. To what extent do other providers in your region/referral network adhere to the guidelines for triage of trauma patients?
2. What are some of the factors that may influence whether other providers follow the guidelines/policies for triage of trauma patients?
3. What, if anything, would you change about trauma triage in your ED?

### Paramedics

My name is _____and I am a researcher from the department of Critical Care Medicine at the University of Pittsburgh. This study is being conducted by Principal Investigator, Dr. Deepika Mohan, Associate Professor of Critical Care Medicine and Surgery, and colleagues to better understand the environment in which you make triage decisions.

I will be asking you several questions about your experience with trauma triage. There are no right answers to these questions. Your responses will be completely confidential and will in no way affect your employment. Although participating in this interview has no direct benefits to you, your responses may help to improve future educational experiences.

We can stop the interview at any point if you feel uncomfortable or do not wish to continue. Also, you do not have to answer any questions that make you feel uncomfortable.

**Do you have any questions at this time? Do I have your permission to audio record our conversation?**

**[Begin recording. State the date, time, and participant identification number]**

**NB: Skip highlighted questions if time is short.**

**First, I would like to learn your thoughts and opinions on guidelines for trauma triage.**

1. Tell me about the guidelines for the transfer of trauma patients in your organization.
   1. Do you know of any evidence that supports the guidelines? (are you aware of any studies or other supporting evidence?)
   2. Do the guidelines impact your decision making in the field?
      1. In your experience, do you think the guidelines change patient outcomes? [Probe: for better, for worse, for non-trauma patients]
   3. How do you find out new information about updates to trauma triage best practices? (peers, supervisors, etc)
2. How are trauma triage guidelines similar to or different from other protocols that exist such as stroke in your region?

**(inner setting) Now, I would like to ask about the infrastructure of your organization related to trauma triage.**

1. What infrastructure is in place to transfer trauma patients? [# of ambulances, staffing, partnerships, logistics, geography]
2. How do you make the decision about which hospital to go to with trauma patients?
   1. What is the impact of the receiving hospital’s capacity?
   2. What are travel implications that may affect the decision (traffic congestion, construction)?
   3. What are your relationships like with the hospitals that you take trauma patients to?
3. Are there norms or expectations within your organization related to trauma triage? (Are there any challenges to upholding these expectations?)

**(outer setting) Now I would like to ask about factors outside of your organization that may impact trauma triage.**

1. About how many ambulance companies are in your region?
   1. Does that influence triage decisions by your organization?
2. Are there any local or state policies that influence triage by your organization?
3. Do you internally review cases of trauma triage?
   1. What kind of feedback do you provide to trauma centers? (Non trauma centers?)
      1. How frequently does that feedback occur?
      2. How does it occur?
   2. What kind of feedback do you receive from trauma centers? (Non trauma centers?)
      1. How often does that happen?

**To wrap up, I have a couple of questions about patient preferences.**

1. To what extent are the preferences of patients and surrogates considered when deciding where to take them after evaluating them at the scene?
2. What are the typical patient and surrogate preferences when you evaluate patients at the scene for an injury?
   1. How often do people decline transport?
   2. How often do people have a preference about which hospital they want to be taken to?
   3. What are some of the circumstances under which patients and surrogates would prefer not to be taken to a trauma center? (What do you do in those circumstances?)

### Patients

My name is _____and I am a researcher from the department of Critical Care Medicine at the University of Pittsburgh. This study is being conducted by Principal Investigator, Dr. Deepika Mohan, Associate Professor of Critical Care Medicine and Surgery, and colleagues to better understand the environment in which doctors make triage decisions about which hospital patients go to for treatment.

I will be asking you several questions about your experiences being hospitalized, whether you were transferred to a different hospital, and your feelings about your experiences. There are no right answers to these questions. Your responses will be completely confidential and will in no way affect the care you receive. Although participating in this interview has no direct benefits to you, your responses may help to improve future patient care experiences.

We can stop the interview at any point if you feel uncomfortable or do not wish to continue. Also, you do not have to answer any questions that make you feel uncomfortable.

**Do you have any questions at this time? Do I have your permission to audio record our conversation?**

**[Begin recording. State the date, time, and participant identification number]**

**First, I would like to learn about your initial injury and arrival at the hospital.**

1. Tell me about how you got a traumatic injury resulting in your hospitalization. [probe for: mechanism and specific injury to the body that was being treated]
2. Could you tell me about your experience in the emergency department?
   1. Which hospital did you go to initially? [Probe: Where located, hospital size]
   2. What other hospitals could you have gone to for your injury? [Probe: Where located, hospital size]
3. Were you admitted to that hospital, or transferred to another hospital for your treatment?
   1. *(if transferred)* How was that decided (or communicated to you)?
      1. What were some of the reasons given for the need to transfer you?
      2. Did your doctor talk to you about the options that were available? Tell me about that.
      3. How much would you have wanted to participate in the decision of where you are treated for your injury?
   2. *(if not transferred/don’t remember)* What was your experience like receiving care at that hospital?
      1. Were you given any option to transfer to another hospital?
      2. Would you have wanted to have the option to transfer to another hospital?
      3. How much would you have wanted to participate in the decision of where you are treated for your injury?
4. How did you feel about being transferred/not being transferred?
   1. What were your priorities about your care and transfer/not being transferred at the time of your injury? *(give an example if needed - some people want to stay near home as top priority, some may have top priority of avoiding pain, some may not want care if it means they cannot have same quality of life...)*
   2. What were your priorities about your care after you were stabilized?

**Next, I have some questions about your time at the hospital and your recovery.**

1. What was that experience like for you?
   1. How long were you in the hospital?
   2. What care did you receive while you were in the hospital? (treatment for injury) (probes: Were you in the ICU? Did you have surgery? What kind of surgery did you have and how many? Did you receive PT or other services?)
2. Do you feel like you have fully recovered from your injury? [back to functional & QoL baseline] [Probe: Do you have any residual issues from your injury?]
   1. How long did it take you to recover?
   2. What has your recovery been like?
      1. Did you go to a rehab facility?
      2. When you went home, did you need any additional medical services? (e.g., nurses, PT, ST, OT)
      3. Did you have other people such as family or friends around to help you?
         1. What things did they help with?

**For my last few questions, I would like to ask you to think about some different scenarios.**

1. In hindsight, do you wish things would have been done differently? *(Do you have any regrets with your care?)*
2. What are some reasons someone might want to get care at a large trauma center?
   1. What are some reasons someone might not want to get care at a large trauma center?

### Surrogates

My name is _____and I am a researcher from the department of Critical Care Medicine at the University of Pittsburgh. This study is being conducted by Principal Investigator, Dr. Deepika Mohan, Associate Professor of Critical Care Medicine and Surgery, and colleagues to better understand the environment in which doctors make triage decisions about which hospital patients go to for treatment.

I will be asking you several questions about your experiences when your loved one was hospitalized, whether they were transferred to a different hospital, and your feelings about the experience. There are no right answers to these questions. Your responses will be completely confidential and will in no way affect the care your loved one receives. Although participating in this interview has no direct benefits to you, your responses may help to improve future patient care experiences.

We can stop the interview at any point if you feel uncomfortable or do not wish to continue. Also, you do not have to answer any questions that make you feel uncomfortable.

**Do you have any questions at this time? Do I have your permission to audio record our conversation?**

**[Begin recording. State the date, time, and participant identification number]**

**First, I would like to learn about the initial injury and arrival at the hospital.**

1. Tell me about how your loved one was injured resulting in their hospitalization. [probe for: mechanism and specific injury to the body that was being treated]
2. Could you tell me about the experience in the emergency department?
   1. Which hospital did your loved one go to initially? [Probe: Where located, hospital size]
   2. What other hospitals could your loved one have gone to for the injury? [Probe: Where located, hospital size]
3. Were they admitted to that hospital, or transferred to another hospital for treatment?
   1. *(if transferred)* How was that decided (or communicated to you)?
      1. What were some of the reasons given for the need to transfer your loved one?
      2. Did your doctor talk to you or your loved one about the options that were available? Tell me about that.
      3. How much would you have wanted to participate in the decision of where they were treated for their injury?
   2. *(if not transferred/don’t remember)* What was the experience like receiving care at that hospital?
      1. Were you given any option to transfer to another hospital?
      2. Would you have wanted to have the option to transfer to another hospital?
      3. How much would you have wanted to participate in the decision of where your loved one was treated for their injury?
4. How did you feel about your loved one being transferred/not being transferred?
   1. What were your priorities about their care and transfer/not being transferred at the time of the injury? *(give an example if needed - some people want to stay near home as top priority, some may have top priority of avoiding pain, some may not want care if it means they cannot have same quality of life...)*
   2. What were your priorities about their care once they were stabilized?

**Next, I have some questions about the time at the hospital and during recovery.**

1. Tell me about your loved one’s hospitalization.
   1. How long were they in the hospital?
   2. What care did they receive while they were in the hospital? (treatment for injury) (probes: Were they in the ICU? Did they have surgery? What kind of surgery did they have and how many? Did they receive PT or other services?)
2. What was the experience like for you of having a loved one in the hospital?
3. Has your loved one fully recovered from their injury? [back to functional & QoL baseline] [Probe: Do they have any residual issues from the injury?]
   1. How long did it take for them to recover?
   2. What has the recovery been like?
      1. Did they go to a rehab facility?
      2. When they went home, did they need any additional medical services? (e.g., nurses, PT, ST, OT)
      3. Were there other people such as family or friends around to help?
         1. What things did they help with?

**For my last few questions, I would like to ask you to think about some different scenarios.**

1. In hindsight, do you wish things would have been *done differently? (Do you have any regrets with your loved one’s care?)*
2. What are some reasons someone might want to get care at a large trauma center?
   1. What are some reasons someone might not want to get care at a large trauma center?

## eFigure Legends

eFigure 1. Trial schematic. Timetable of trial activities and organizational schema

eFigure 2. Schematic of behavioral intervention development. Outline of steps taken to develop *Night Shift 2024.*
